# Prevalence and severity of atrial cardiomyopathy in patients with recently diagnosed atrial fibrillation and stroke risk factors and its association with early rhythm control: a secondary analysis of EAST-AFNET 4

**DOI:** 10.1093/europace/euaf256

**Published:** 2025-10-08

**Authors:** Andreas Goette, Marc D Lemoine, Katrin Borof, Ulrich Schotten, Günter Breithardt, A John Camm, Harry J G M Crijns, Lars Eckardt, Andreas Metzner, Stephan Willems, Antonia Zapf, Renate B Schnabel, Larissa Fabritz, Paulus Kirchhof

**Affiliations:** Department of Cardiology and Intensive Care Medicine, St. Vincenz Hospital, Am Busdorf 2, 33098 Paderborn, Germany; Medical Faculty, Otto-von-Guericke University, Leipziger Str. 44, 39120 Magdeburg, Germany; AFNET e.V., Mendelstr. 11, 48149 Münster, Germany; AFNET e.V., Mendelstr. 11, 48149 Münster, Germany; Department of Cardiology, University Heart and Vascular Center Hamburg, University Medical Center Hamburg Eppendorf, Martinistr. 52, 20251 Hamburg, Germany; AFNET e.V., Mendelstr. 11, 48149 Münster, Germany; Department of Cardiology, University Heart and Vascular Center Hamburg, University Medical Center Hamburg Eppendorf, Martinistr. 52, 20251 Hamburg, Germany; Department of Cardiology, Maastricht University Medical Centre and Cardiovascular Research Institute Maastricht, P.Debyelaan 25, 6229 HX Maastricht, The Netherlands; Department of Cardiology and Angiology, University Hospital Münster and AFNET e.V., Albert-Schweitzer-Campus 1, 48149 Münster, Germany; Cardiology Clinical Academic Group, Molecular and Clinical Sciences Research Institute, City St. George’s University of London, Cranmer Terrace, London SW17 0RE, UK; Department of Cardiology, Maastricht University Medical Centre and Cardiovascular Research Institute Maastricht, P.Debyelaan 25, 6229 HX Maastricht, The Netherlands; Department of Cardiology and Angiology, University Hospital Münster and AFNET e.V., Albert-Schweitzer-Campus 1, 48149 Münster, Germany; AFNET e.V., Mendelstr. 11, 48149 Münster, Germany; Department of Cardiology, University Heart and Vascular Center Hamburg, University Medical Center Hamburg Eppendorf, Martinistr. 52, 20251 Hamburg, Germany; Asklepios Klinik St. Georg, Klinik für Kardiologie und internistische Intensivmedizin, Lohmühlenstr. 5, 20099 Hamburg, Germany; Institute for Medical Biometry and Epidemiology, University Hospital Hamburg-Eppendorf, Martinistr. 52, 20251 Hamburg, Germany; AFNET e.V., Mendelstr. 11, 48149 Münster, Germany; Department of Cardiology, University Heart and Vascular Center Hamburg, University Medical Center Hamburg Eppendorf, Martinistr. 52, 20251 Hamburg, Germany; AFNET e.V., Mendelstr. 11, 48149 Münster, Germany; Department of Cardiology, University Heart and Vascular Center Hamburg, University Medical Center Hamburg Eppendorf, Martinistr. 52, 20251 Hamburg, Germany; AFNET e.V., Mendelstr. 11, 48149 Münster, Germany; Department of Cardiology, University Heart and Vascular Center Hamburg, University Medical Center Hamburg Eppendorf, Martinistr. 52, 20251 Hamburg, Germany

**Keywords:** atrial cardiomyopathy, Atrial fibrillation, Atrial size, Biomarkers, Echocardiography, Outcome

## Abstract

**Aims:**

Observational data suggest that atrial cardiomyopathy can precede the clinical diagnosis of atrial fibrillation (AF) and that severe forms of atrial cardiomyopathy render rhythm control therapy futile. The aim was to quantify atrial cardiomyopathy in patients with recently diagnosed AF and to determine possible interactions between atrial cardiomyopathy and early rhythm control therapy in the EAST-AFNET 4 trial.

**Methods and results:**

This prespecified analysis of the EAST-AFNET 4 trial quantified baseline atrial cardiomyopathy using left atrial (LA) size, PR interval, and NT-proBNP. Outcomes were compared between atrial cardiomyopathy categories. Interactions between early rhythm control, the randomized therapy in EAST-AFNET 4, and atrial cardiomyopathy were determined. Outcomes included the primary outcome of EAST-AFNET 4 (cardiovascular death, stroke, hospitalization for heart failure or acute coronary syndromes), recurrent AF, and safety outcomes (serious adverse events of special interest or all-cause death). In an exploratory analysis, angiopoietin-2 (ANGPT2) as well as bone morphogenetic protein 10 (BMP10) were assessed to predict atrial cardiomyopathy. Most patients showed signs of atrial cardiomyopathy at baseline [69% with at least mildly elevated LA size, 23% with prolonged PR interval (≥200 ms), 56% with NT-proBNP > 365 pg/mL]. Severe atrial cardiomyopathy, defined as the highest tertile of LA size, PR interval, and NT-proBNP, was associated with higher rates of first primary outcome [HR 7.97 (2.32, 27.37); *P* < 0.001]. Early rhythm control was effective with and without atrial cardiomyopathy (*P*_interaction_ = 0.160). While ANGPT2 levels showed an association to LA diameter and to atrial cardiomyopathy severity/stage, BMP 10 was not associated with atrial cardiomyopathy.

**Conclusion:**

Most patients have signs of atrial cardiomyopathy in the first year after AF diagnosis. Patients with advanced stages of atrial cardiomyopathy had a higher rate of primary outcome events and more recurrent AF. Nevertheless, early rhythm control therapy retains its efficacy across the spectrum of atrial cardiomyopathy severities. Consequently, atrial cardiomyopathy severity should not be a reason to withhold rhythm control therapy.

**Condensed Abstract:**

This prespecified analysis of the EAST-AFNET 4 trial used baseline left atrial diameter, PR interval, and NT-proBNP to quantify atrial cardiomyopathy in patients with recently diagnosed AF. Outcome rates were compared between atrial cardiomyopathy categories, and interactions between atrial cardiomyopathy and early rhythm-control were determined. Most patients had atrial cardiomyopathy (84% with enlarged left atria). Patients with advanced atrial cardiomyopathy had higher rates of primary outcome during follow-up. Early rhythm control was effective with and without atrial cardiomyopathy (*P*_interaction_ = 0.160).

## Graphical Abstract

**Graphical Abstract:**
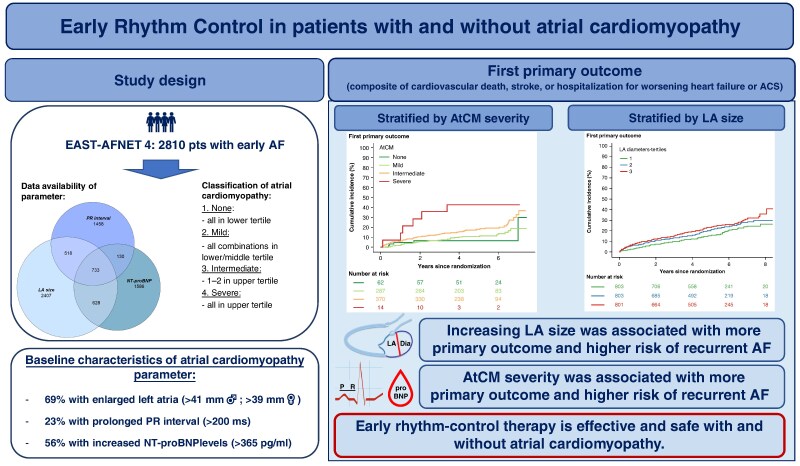
Impact of atrial cardiomyopathy (AtCM) defined by a composite of structural changes, electrophysiological parameters, and blood biomarkers on clinical outcome in patients with recently diagnosed atrial fibrillation (AF). There is a gradual interaction between the degree of AtCM with occurrence of primary outcome and AF recurrence. Worst outcome and highest rates of AF recurrence were observed in patients in the third tertile of AtCM changes. Of note, early rhythm control was effective even in AF patients with most pronounced AtCM to reduce primary clinical outcome.

What’s new?

- This prespecified analysis of the EAST-AFNET 4 trial used baseline left atrial diameter, PR interval, and NT-proBNP to quantify atrial cardiomyopathy in patients with recently diagnosed AF.
- Outcome rates were compared between atrial cardiomyopathy categories, and interactions between atrial cardiomyopathy and early rhythm control were determined.
- Most patients had atrial cardiomyopathy (84% with enlarged left atria). Patients with advanced atrial cardiomyopathy had higher rates of primary outcome during follow-up.
- Early rhythm control was effective with and without atrial cardiomyopathy.

## Introduction

Atrial cardiomyopathy can be defined as ‘any complex of structural, architectural, contractile, or electrophysiological changes affecting the atria with the potential to produce clinically relevant manifestations’.^1^ Detection of atrial cardiomyopathy and quantification of its severity rely on integration of parameters from ECG, cardiac imaging, and blood biomarkers.^2^ Severe forms of atrial cardiomyopathy can render rhythm control therapy more difficult or futile. Clinicians frequently withhold rhythm control therapy in patients with advanced atrial cardiomyopathy.^3^

While rhythm control therapy may be less often successful in patients with advanced atrial cardiomyopathy, data on its efficacy and safety in patients with different degrees of atrial cardiomyopathy are scarce. Importantly, a potential interaction between atrial cardiomyopathy and rhythm control therapy has never been tested. This prespecified secondary analysis of the EAST-AFNET 4 trial therefore determined whether atrial cardiomyopathy interacts with rhythm control therapy and whether atrial cardiomyopathy categories alter the risk of cardiovascular events in patients with AF.

## Methods

**Figure 1:**
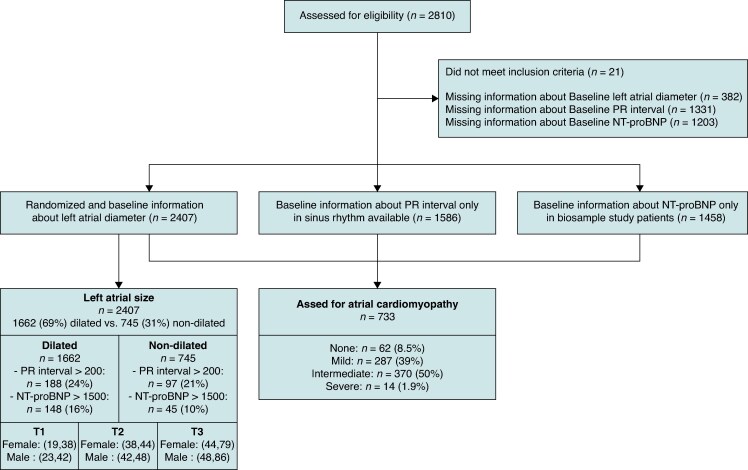
Consort diagram.

The design of the EAST-AFNET 4 trial and its main results have been published.^4,5^ The trial population consisted of adults (≥18 years of age) who had recently diagnosed atrial fibrillation diagnosed ≤12 months before enrollment, and who were older than 75 years of age, had had a previous transient ischemic attack or stroke, or met two of the following criteria: age > 65 years, female sex, heart failure, hypertension, diabetes mellitus, severe coronary artery disease, chronic kidney disease [Modification of Diet in Renal Disease stage 3 or 4 (glomerular filtration rate, 15–59 mL per minute per 1.73 m^2^ of body-surface area)], and left ventricular hypertrophy (diastolic septal wall width, >15 mm). For this prespecified analysis, patients entering the trial were grouped and analysed in accordance to early rhythm control vs. usual care. Clinical features, left atrial (LA) diameter, and PR interval were recorded in all patients at baseline (*Figure 1*). In a subcohort (*n* = 1589), blood samples were taken at baseline and circulating biomarkers ANGPT-2, BMP-10, and NT-proBNP were centrally quantified (Roche, Penzberg, Germany). Patients were followed over a mean period of 5.1 years per patient.

Outcomes included the first primary outcome of EAST-AFNET 4 (cardiovascular death, stroke, hospitalization for heart failure, or acute coronary syndromes), recurrent AF, and safety outcomes (serious adverse events of special interest or all-cause death). Interaction between atrial cardiomyopathy parameters and first primary outcome were assessed. The secondary primary outcome of EAST-AFNET 4, nights spent in hospital, was not different between randomized groups. It was therefore not analysed.

The sponsor is AFNET, Münster, Germany. The protocol was approved by ethical review in Münster (Germany) and boards for all institutions.^6,7^ Trial reg: ISRCTN04708680, NCT01288352, EudraCT2010-021258-20).

### Statistical methods

Clinical characteristics and atrial cardiomyopathy parameters of the AF patients are presented as mean and standard deviation, median and IQR, or number and percentage. For comparison of groups, *P*-values resulting from mixed linear regression models for metric variables and mixed logistic regression models for binary categorical variables, and Analysis of Deviance Table (Type II Wald χ^2^ tests) were calculated. Site was included as random effect. For multinomial categorical variables, a random effect was not included.

The treatment effects were determined in each group. Cox regression models with an interaction term between treatment group and atrial cardiomyopathy parameters and site as a shared frailty term were estimated for the first primary outcome and its individual components (cardiovascular death, stroke, hospitalization for worsening of heart failure, and hospitalization for ACS) and recurrent AF. The resulting estimates are expressed as hazard ratios with 95% confidence interval. All interaction *P*-values were calculated with likelihood ratio test, and values of *P* < 0.05 were considered statistically significant. Analyses were performed using R software, version 4.4.2 (R Project for Statistical Computing).

## Results

**Figure 2:**
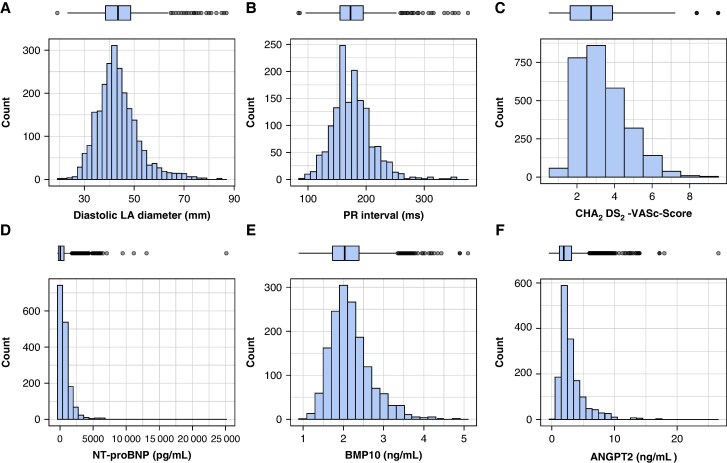
Distribution of biomarkers: distribution of left atrial size (diastolic left atrial diameter), PR interval, NT-proBNP, and the novel atrial markers BMP10 and ANGPT2 in the study population illustrated by bar chart (lower part) and box plot (upper part).

Parameters of atrial cardiomyopathy, including LA size, NT-proBNP, and PR interval, varied widely in the population, reflecting the range (severity) of atrial cardiomyopathy found in patients with recently diagnosed AF (*Figure 1*, *Figure 2*). The majority of patients had some degree of atrial cardiomyopathy, in particular LA dilatation (see Supplementary material online, *Table S1*). Left atrial size was low normal in 31% of individuals at baseline (<41 mm in men or <39 mm in women). Twenty-three percentage of all AF patients had a PR interval ≥200 ms at baseline, and 56% showed increased levels of NT-proBNP (>365 pg/mL). Levels of blood biomarkers are listed in *Table 1* (summary table for all atrial cardiomyopathy parameters as mean + SD in the total cohort, early rhythm control, and usual care).

**Table 1:** Characteristics of atrial cardiomyopathy in early rhythm control and usual care

Characteristic	Overall *N* = 2789	Treatment group		*P*-value	Unknown (overall)
		Early rhythm control *N* = 1395 (50%)	Usual care *N* = 1394 (50%)		
LA diameter (mm)				0.42	382
Mean ± SD	44 ± 8	44 ± 8	44 ± 9		
Median (Q1, Q3)	43 (38, 48)	43 (38, 48)	43 (39, 48)		
PR interval (ms)				0.21	1331
Mean ± SD	177 ± 35	176 ± 32	178 ± 37		
Median (Q1, Q3)	174 (156, 196)	172 (156, 190)	176 (156, 200)		
NT-proBNP				0.29	1203
Mean ± SD	798 ± 1203	772 ± 1268	825 ± 1135		
Median (Q1, Q3)	452 (182, 1001)	441 (175, 966)	467 (187, 1036)		
BMP10				0.93	1203
Mean ± SD	2.19 ± 0.52	2.19 ± 0.54	2.19 ± 0.50		
Median (Q1, Q3)	2.11 (1.83, 2.43)	2.10 (1.82, 2.41)	2.11 (1.83, 2.45)		
ANGPT2				0.82	1203
Mean ± SD	3.22 ± 2.24	3.24 ± 2.29	3.20 ± 2.20		
Median (Q1, Q3)	2.53 (1.87, 3.71)	2.53 (1.87, 3.66)	2.53 (1.87, 3.76)		
CHA_2_DS_2_-VASc score				0.73	
Mean ± SD	3.35 ± 1.31	3.36 ± 1.30	3.34 ± 1.32		
Median (Q1, Q3)	3.00 (2.00, 4.00)	3.00 (2.00, 4.00)	3.00 (2.00, 4.00)		

ANGPT2, antiopoietin-2; BMP10, bone morphogenetic protein 10; LA, left atrium;.

Left atrial size was available in 2407/2789 patients (86%). PR interval could only be measured in 1485/2789 patients (53%) in sinus rhythm at baseline. Blood biomarkers were available in 1589 patients donating blood samples (57%,^5,8^). To minimize missing values, LA size was used as the primary feature for atrial cardiomyopathy in analyses related to outcomes.

### Single parameter of atrial cardiomyopathy

#### Left atrial size

**Figure 3:**
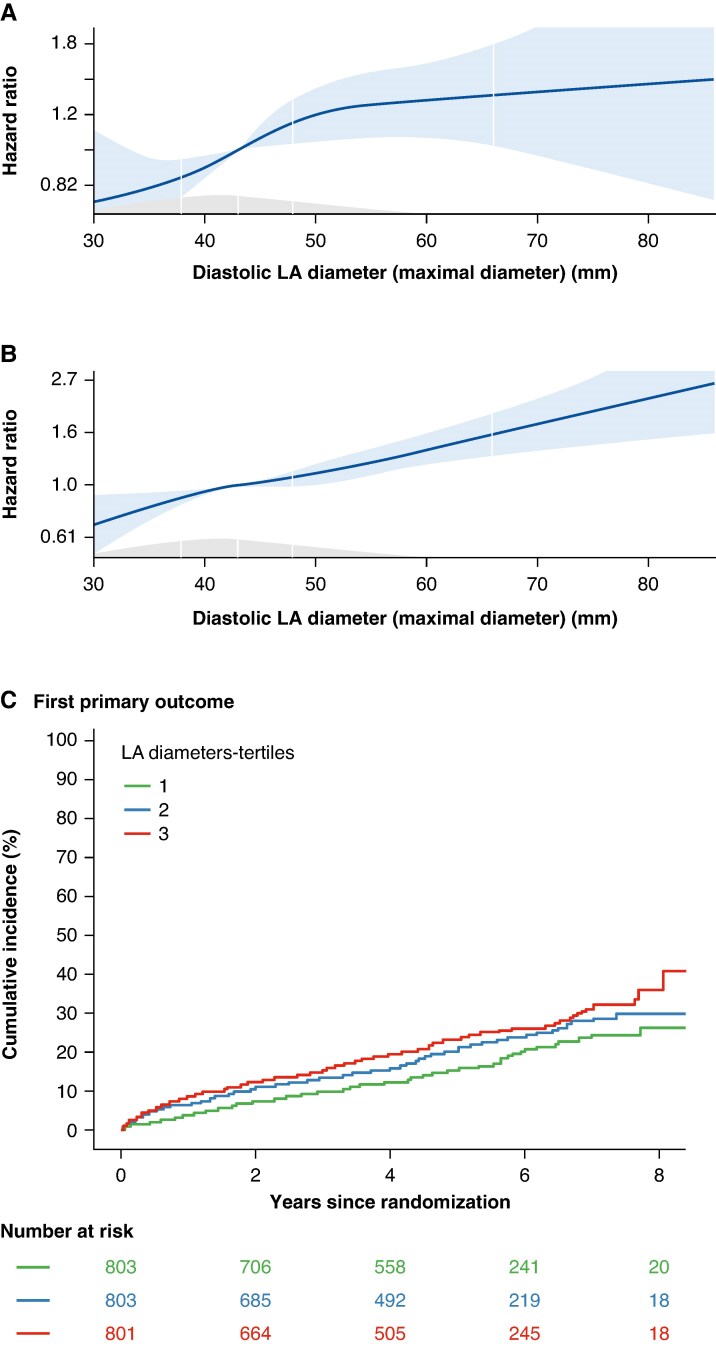
LA size and outcome. First primary outcome (*A*), a composite of cardiovascular death, stroke, or unplanned hospitalization for heart failure or acute coronary syndrome, and recurrent AF (*B*) is associated with diastolic LA diameter shown as hazard ratio (HR) from Cox regression in this study population. (*C*) Aalen–Johnsen cumulative curves for first primary outcome.

Overall, the risk for a cardiovascular death, stroke, or unplanned hospitalization for heart failure or ACS increased with increasing LA size (HR 1.25 [1.10, 1.41], *P* < 0.001; *Figure 3A* and *C*). In parallel, increasing LA size was associated with a higher risk of recurrent AF (HR 1.17 [1.08, 1.27], *P* < 0.001; *Figure 3B*). Upon cox regression analysis, LA size remained an independent predictor of cardiovascular events (*Table 2*, *Figure 3C*). Early rhythm control remained effective across all LA sizes in reducing the first primary outcome (*P*_interaction_ = 0.616) and recurrent AF (*P*_interaction_ = 0.178).

**Table 2:** Predictors of the primary outcome, a composite of cardiovascular death, stroke, or unplanned hospitalization for heart failure or acute coronary syndrome

Variable	HR per SD	95% CI	*P* value
Early rhythm control	0.75	[0.63, 0.89]	0.001
Diastolic LA diameter (mm)	1.19	[1.08, 1.32]	0.001
Sex: Male	1.03	[0.85, 1.25]	<0.001
Age	1.47	[1.33, 1.63]	0.030
LVEF < 50%	1.33	[1.03, 1.71]	0.209
NYHA class I (ref = 0)	1.2	[0.9, 1.6]	<0.001
NYHA class II (ref = 0)	1.7	[1.34, 2.18]	<0.001
NYHA class III (ref = 0)	2.49	[1.71, 3.61]	<0.001
CAD	1.58	[1.28, 1.95]	0.008
Diabetes mellitus	1.31	[1.07, 1.6]	0.002

Hazard ratios are calculated using cox regression including random group, sex, age, diastolic LA diameter and all clinical features associated with the primary outcome in the main analysis of the EAST-AFNET 4 trial.

#### PR interval

PR interval was measured in 1485 patients who were in sinus rhythm at baseline. These patients were used for further analyses. Overall, PR interval was associated with the primary outcome (HR 1.23 [1.06, 1.42] *P* = 0.006; Supplementary material online, *Figure S1A*) but not associated with recurrent AF (HR 1.07 [0.96, 1.20], *P* = 0.234; Supplementary material online, *Figure S1B*). A longer PR interval did not interact with the efficacy of early rhythm control therapy concerning primary outcome (*P*_interaction_ = 0.421) or recurrent AF (*P*_interaction_ = 0.509).

#### NT-proBNP

NT-proBNP was centrally quantified in 1589 patients.^8,9^ Median NT-proBNP was 452 (182, 1001) pg/mL. Increasing NT-proBNP concentrations were associated with a higher risk of cardiovascular events. Overall, NT-proBNP was associated with the primary outcome (HR 1.81 [1.53, 2.15] *P* < 0.001; Supplementary material online, *Figure S2A*) and with recurrent AF (HR 1.52 [1.37, 1.69] *P* = 0.234; Supplementary material online, *Figure S2B*). A higher NT-proBNP did not interact with the efficacy of early rhythm control therapy concerning primary outcome (*P*_interaction_ = 0.421).

#### Atrial cardiomyopathy

**Figure 4:**
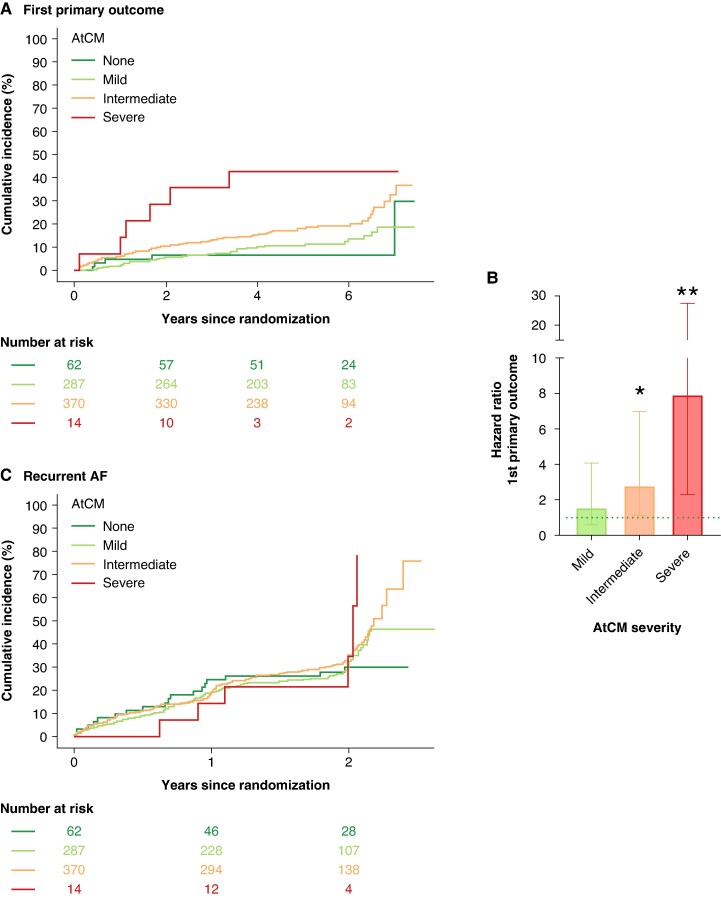
(*A*) Aalen–Johnsen cumulative curves for first primary outcome dependent on severity of atrial cardiomyopathy (AtCM). (*B*) Bar chart of hazard ratio for first primary outcome of severity classes of atrial cardiomyopathy (AtCM), no atrial cardiomyopathy was used as a reference (dotted line). (*C*) Aalen–Johnsen cumulative curves for recurrent AF dependent on severity of atrial cardiomyopathy.

To further explore a possible effect of advanced atrial cardiomyopathy on outcomes, patients with clear signs of atrial cardiomyopathy in all three parameters were identified by selecting patients in the highest tertiles. As LA size differs between men and women, sex-specific tertiles were determined. The cut-off of the highest tertile was 44 mm (women) and 48 mm (men) for LA diameter, 187 ms for PR interval, and >777 pg/mL for NT-proBNP. There were 14 patients with severe atrial cardiomyopathy, defined as the upper tertile in all three atrial cardiomyopathy parameters (*Table 3*). They were older and had more comorbidities than patients with less severe atrial cardiomyopathy (*Table 4*). Patients with intermediate form of atrial cardiomyopathy (one or two parameters in the upper tertile) or mild form (any parameter in low or middle tertile) showed intermediate phenotypes (*Table 4*). As expected, patients with severe atrial cardiomyopathy (HR 7.97 (2.32, 27.37); *P* < 0.001, *n* = 14) and with intermediate atrial cardiomyopathy (HR 2.8 (1.12, 6.99); *P* = 0.027, *n* = 370) had a higher rate of primary outcome events and more recurrent AF (*Figure 4*) than patients with no atrial cardiomyopathy (*n* = 62), whereas mild atrial cardiomyopathy (HR 1.59 (0.62, 4.08); *P* = 0.339, *n* = 287) was not different. Early rhythm reduced cardiovascular complications (first primary outcome) regardless of the level of baseline atrial cardiomyopathy parameters during a 5.1-year follow-up (early rhythm control HR 0.71 (0.49–1.03); interaction *P* = 0.160, *Table 4*). To emphasize that early rhythm control is effective across all groups of atrial cardiomyopathy to reduce the first primary outcome, we found no significant interaction between the subgroups of mild atrial cardiomyopathy (HR 0.31 [0.03, 3.08], *P* = 0.317) and intermediate/severe atrial cardiomyopathy (HR 0.18 [0.02, 1.73], *P* = 0.138).

**Table 3:** Case numbers of patient stratified by atrial cardiomyopathy severity^a^

	Randomization group		Total
	Early rhythm control	Usual care	
Atrial cardiomyopathy severity			
None	38 (10%)	24 (6.7%)	62 (8.5%)
Mild	154 (41%)	133 (37%)	287 (39%)
Intermediate	177 (47%)	193 (54%)	370 (50%)
Severe	4 (1.1%)	10 (2.8%)	14 (1.9%)
Total	373 (100%)	360 (100%)	733 (100%)

^a^Classification of atrial cardiomyopathy by LA-diameter, NT-proBNP, and PR-interval: none: all parameter in lower tertile; mild: all combination in lower and middle tertile; intermediate: one or two parameter in the upper tertile; severe: all parameter in upper tertile.

**Table 4:** Efficacy outcomes per treatment group and atrial cardiomyopathy severity^a^

	Early rhythm control				Usual care				*P*-value interaction
	None	Mild	Intermediate	Severe	None	Mild	Intermediate	Severe	
First primary outcome—events/person-yr (incidence/100 person-yr)	4/182 (2.2)	19/756 (2.5)	28/810 (3.5)	0/21 (0)	1/136 (0.7)	16/614 (2.6)	46/875 (5.3)	6/27 (22.4)	0.160
Components of first primary outcome—events/person-yr (incidence/100 person-yr)									
Death from cardiovascular causes	2/193 (1)	4/793 (0.5)	9/872 (1)	0/21 (0)	1/136 (0.7)	2/651 (0.3)	16/963 (1.7)	1/45 (2.2)	
Stroke	1/187 (0.5)	6/779 (0.8)	4/858 (0.5)	0/21 (0)	0/136 (0)	0/651 (0)	10/931 (1.1)	1/43 (2.3)	
Hospitalization with worsening of HF	1/188 (0.5)	7/777 (0.9)	13/847 (1.5)	0/21 (0)	0/136 (0)	8/641 (1.2)	19/913 (2.1)	5/29 (17.5)	
Hospitalization with ACS	2/193 (1)	4/784 (0.5)	9/843 (1.1)	0/21 (0)	0/136 (0)	7/625 (1.1)	8/946 (0.8)	0/45 (0)	
Other									
At least one recurrent AF	9/146 (6.2)	52/577 (9)	57/638 (8.9)	2/13 (15.9)	9/90 (10)	45/481 (9.4)	83/614 (13.5)	4/29 (13.9)	

ACS, acute coronary syndrome.

^a^Classification of atrial cardiomyopathy by LA-diameter, NT-proBNP, and PR-interval: none: all parameter in lower tertile; mild: all combination in lower and middle tertile; intermediate: one or two parameter in the upper tertile; severe: all parameter in upper tertile.

Since PR interval was not available in 47% of patients due to AF at baseline characterization and since it might be influenced by antiarrhythmic drugs, we performed a second analysis including only LA size and NT-proBNP as parameter for atrial cardiomyopathy. There were 184 patients (14%) with severe atrial cardiomyopathy in upper tertile of LA size and NT-proBNP (see Supplementary material online, *Table S2*). These patients more comorbidities than patients with less severe atrial cardiomyopathy, including higher body mass index, lower LV-ejection fraction, higher NYHA class, and more persistent atrial fibrillation (see Supplementary material online, *Table S3*). Interestingly, in comparison to the analysis with all three parameter, patient with severe atrial cardiomyopathy based on LA size and NT-proBNP received more often ablation therapy compared to all other severities of atrial cardiomyopathy (see Supplementary material online, *Table S4*). The primary outcome and recurrent AF (see Supplementary material online, *Figure S6*, Supplementary material online, *Tables S5* and *S6*) occurred more often in patients with severe atrial cardiomyopathy. Early rhythm reduced cardiovascular complications (first primary outcome) regardless of the level of baseline atrial cardiomyopathy parameters during a 5.1-year follow-up (early rhythm control HR 0.69 (0.45–0.88); interaction *P* = 0.235).

#### Atrial cardiomyopathy in paroxAF and persAF

Patients with persAF had more severe signs of atrial cardiomyopathy than patients with paroxAF (*Table 5*) including larger left atria (persAF 48 ± 10 mm, paroxAF 42 ± 7 mm, *P*-value < 0.001), higher NT-proBNP (persAF 1193 ± 1609 pg/mL, paroxAF 518 ± 772 pg/mL, *P*-value < 0.001) and also longer PR intervals for those patients presenting in sinus rhythm at baseline (persAF 184 ± 38 ms, paroxAF 176 ± 35 ms, *P*-value = 0.007).

**Table 5:** Characteristics of atrial cardiomyopathy in paroxysmal and persistent atrial fibrillation

Characteristic	Overall *N* = 1737^a^	AF type		*P*-value^b^	Unknown (overall)
		Paroxysmal *N* = 994 (57%)^a^	Persistent *N* = 743 (43%)^a^		
LA diameter (mm)				<0.001	220
Mean ± SD	44 ± 9	42 ± 7	48 ± 10		
Median (Q1, Q3)	43 (39, 49)	41 (37, 46)	46 (42, 52)		
PR interval				0.007	835
Mean ± SD	178 ± 35	176 ± 35	184 ± 38		
Median (Q1, Q3)	175 (156, 196)	173 (156, 192)	180 (160, 200)		
NT-proBNP				<0.001	711
Mean ± SD	805 ± 1246	518 ± 772	1193 ± 1609		
Median (Q1, Q3)	491 (195, 1049)	278 (130, 605)	846 (478, 1463)		
BMP10				<0.001	711
Mean ± SD	2.19 ± 0.52	2.10 ± 0.47	2.32 ± 0.56		
Median (Q1, Q3)	2.11 (1.83, 2.43)	2.04 (1.77, 2.33)	2.20 (1.95, 2.60)		
ANGPT2				<0.001	711
Mean ± SD	3.28 ± 2.29	2.80 ± 2.09	3.93 ± 2.38		
Median (Q1, Q3)	2.57 (1.90, 3.86)	2.24 (1.75, 3.01)	3.33 (2.29, 4.84)		
CHA_2_DS_2_-VASc score				0.34	
Mean ± SD	3.36 ± 1.33	3.33 ± 1.33	3.38 ± 1.32		
Median (Q1, Q3)	3.00 (2.00, 4.00)	3.00 (2.00, 4.00)	3.00 (2.00, 4.00)		

ANGPT2, antiopoietin-2; BMP10, bone morphogenetic protein 10; LA, left atrium;.

^a^Mean (SD) and median (IQR).

^b^ *P*-values resulting from mixed linear regression models.

#### Exploratory biomarker

In an exploratory analysis of biomarker, angiopoietin-2 (ANGPT2), a novel candidate biomarker of endothelial inflammation and vascular remodelling showed an association to LA diameter and to atrial cardiomyopathy severity/stage (*Table 6*) while bone morphogenetic protein 10 (BMP10), which is regulated by PITX2 gene, did not show an association.

**Table 6:** Exploratory analysis of biomarker depending on (A) LA diameter and (B) atrial cardiomyopathy atrial cardiomyopathy severity

Characteristic	LA diameter—tertiles		
	1 *N* = 803^a^ (missing *n* = 340)	2 *N* = 802^a^ (missing *n* = 319)	3 *N* = 802^a^ (missing *n* = 387)
ANGPT2 (ng/mL)	2.25 (1.69, 3.28)	2.58 (1.85, 3.76)	2.90 (2.10, 4.45)
BMP10 (ng/mL)	2.09 (1.85, 2.46)	2.10 (1.84, 2.41)	2.13 (1.82, 2.44)

^a^Median (Q1, Q3).

**Table:** 

Characteristic	Atrial cardiomyopathy			
	None *N* = 62^a^	Mild *N* = 287^a^	Intermediate *N* = 370^a^	Severe *N* = 14^a^
ANGPT2 (ng/mL)	1.95 (1.39, 2.42)	2.00 (1.58, 2.68)	2.29 (1.75, 3.12)	2.79 (2.25, 3.53)
BMP10 (ng/mL)	2.01 (1.76, 2.23)	2.03 (1.79, 2.30)	2.01 (1.76, 2.32)	2.02 (1.52, 2.58)

ANGPT2, angiopoietin-2; BMP10, bone morphogenetic potein.

^a^Median (Q1, Q3).

### Safety analysis

The numbers of patients with a primary-safety-outcome event did not differ between the treatment groups (early rhythm control and usual care) in the corresponding group of atrial cardiomyopathy (*Table 7*), in parallel to the safety analysis dependent on LA size (see Supplementary material online, *Table S1*). There was neither a significant interaction between the primary composite safety outcome with severity of atrial cardiomyopathy (*P*-interaction 0.407, *Table 6*) nor with for LA size (*P*-interaction = 0.911, Supplementary material online, *Table S1*).

**Table 7:** Safety outcomes stratified by severity of atrial cardiomyopathy and treatment group

	Early rhythm control				Usual care				*P*-value interaction
	None	Mild	Intermediate	Severe	None	Mild	Intermediate	Severe	
*n*	38	154	177	4	24	133	193	10	
Primary composite safety outcome	3 (7.9)	22 (14.3)	32 (18.1)	0 (0.0)	1 (4.2)	12 (9.0)	35 (18.1)	2 (20.0)	0.407
Stroke	1 (2.6)	6 (3.9)	4 (2.3)	0 (0.0)	0 (0.0)	0 (0.0)	10 (5.2)	1 (10.0)	
Death	2 (5.3)	10 (6.5)	19 (10.7)	0 (0.0)	1 (4.2)	12 (9.0)	24 (12.4)	1 (10.0)	0.875
Serious adverse event of special interest related to rhythm control therapy	0 (0.0)	7 (4.5)	12 (6.8)	0 (0.0)	0 (0.0)	0 (0.0)	3 (1.6)	0 (0.0)	

## Discussion

### Main findings

This prespecified subanalysis of the EAST-AFNET 4 trial yielded two main findings.

1. Patients with recently diagnosed AF present with a range of atrial cardiomyopathy severities from no detectable atrial cardiomyopathy (8.5%) to severe forms with enlarged atria, high NT-proBNP concentrations, and long PR intervals. Patients with severe stages of atrial cardiomyopathy had a higher rate of primary outcome events and more recurrent AF.
2. Early rhythm control therapy retains its efficacy with and without atrial cardiomyopathy. Thus, atrial cardiomyopathy stage should not be a reason to withhold early rhythm control therapy.

It has been shown that the CHA_2_DS_2_-VASc score is helpful to predict cardiovascular events in patients with AF. In the line of evidence, a previous EAST-AFNET substudy by Rillig *et al.* analysed 1093 patients with CHA_2_DS_2_-VASc score ≥4 and 1696 with CHA_2_DS_2_-VASc score <4. Early rhythm control reduced the composite primary efficacy outcome of cardiovascular death, stroke, or hospitalization for worsening of heart failure or for acute coronary syndrome in patients with CHA_2_DS_2_-VASc score ≥4, but not in patients with CHA_2_DS_2_-VASc score <4.^9^ When female sex was ignored for the creation of higher and lower risk groups (CHA_2_DS_2_-VA score), the primary safety outcome remained significant. Thus, early rhythm control is effective in AF patients with CHA_2_DS_2_-VASc score ≥4 to reduce cardiovascular outcomes.^10^

The concept of atrial cardiomyopathy was systematically introduced in 2016 as an expert consensus document. Atrial cardiomyopathy was defined as ‘any complex of structural, architectural, contractile or electrophysiological changes affecting the atria with the potential to produce clinically relevant manifestations’.^11^ Based on this definition, the present analysis combined imaging, ECG and blood biomarkers in the present analysis to detect atrial cardiomyopathy. The updated consensus document aims to define three stages of atrial cardiomyopathy. We used a tertile-stratification of this real-world cohort of patients with early diagnosis of atrial fibrillation, in order to create unbiased categories to investigate interaction with therapy effects. This definition cannot replace literature-based stratifications including echocardiography.^1,12^ However, this tertile-based stratification might help to contextualize atrial cardiomyopathy severity also in patients with a longer history of atrial fibrillation. In addition, we assessed the effect of early rhythm control in relation to atrial cardiomyopathy stages.

It is known that changes in LA diameter^13^ and increased concentrations of NT-proBNP^6,7^ are associated with higher rates of AF incidence as well as AF recurrence after cardioversion or catheter ablation.^14^ Left atrial size can be a reflection of advanced cardiovascular disease, with heart failure and advanced disease of the AV valves leading to LA dilation. This analysis suggests that early rhythm-control therapy is effective and safe in the presence of severely dilated left atria. Atrial dilation can lead to altered expression of adhesion molecules and inflammatory markers at the endocardial surface.^1,11^ Furthermore, increased atrial pressure and volume overload induced hypertrophy of atrial myocytes, collagen accumulation in the interstitial matrix, and atrial fibrosis.^15^ These structural changes aggravate atrial conduction slowing, which is reflected by a prolonged PR interval. Atrial stiffness induces increased ventricular wall stress and NT-pro-BNP release. Vice versa, patients with pre-existiing heart failure and high NT-proBNP levels might accelerate the development of atrial cardiomyopathy due to volume load. In addition, episodes of AF, in particular longer-lasting AF episodes, appear to accelerate structural and electrophysiological changes in the atrial tissue. This concept helps to explain the finding of the present analysis that atrial cardiomyopathy was more pronounced in patients with persAF compared to paroxAF. A previous EAST-AFNET analysis showed that the AF pattern influences outcomes: Chronic paroxAF and persAF were more linked to stroke and heart failure compared to first-diagnosed AF, which was related to ACS.^16^ Similar to this analysis, early rhythm-control was effective across AF patterns in that analysis.^16^

### Elevated NT-proBNP and concentrations of ANGPT2 and BMP10

A previous analysis of the EAST AFNET biosample study found that low concentrations of ANGPT2, BMP10, and NT-proBNP identify patients with AF who are likely to attain sinus rhythm during follow-up.^8^ Based on these findings, NT-proBNP concentrations were used to quantify Atrial cardiomyopathy in this study. In addition, a novel result is the presence of elevated concentrations of ANGPT2 in AF patients with atrial cardiomyopathy. ANGPT2 is a prominent marker of AF in the UK Biobank.^17^ Of note, ANGPT2 levels increased with atrial cardiomyopathy severity in the present analysis. In contrast, BMP10, which is considered to be an atrial-specific marker controlled by PITX2,^18,19^ did not change with Atrial cardiomyopathy severity. This finding is of interest, since previous studies showed that systemic BMP10 levels are related to cardiovascular events and recurrent AF.^8,9,20–22^ Overall, in this exploratory analysis, ANGPT2, in contrast to BMP10, may therefore be useful to estimate atrial cardiomyopathy severity in addition to NT-proBNP.

#### Clinical implication

The present study assessed the impact of atrial cardiomyopathy parameters on outcome in patients recently diagnosed atrial fibrillation. The third tertile of each individual parameter (PQ interval, LA diameter, and NTproBMP) was used to define the severity of atrial cardiomyopathy. Thus, among patients with recently diagnosed AF, the present results are helpful in clinical practice to characterize a high-risk group with worse outcomes. Further research is warranted to explore the role of atrial cardiomyopathy in selecting rhythm control in patients with long-standing AF, especially long-standing persistent AF. Nevertheless, our study shows for the first time that early rhythm control is even helpful in patients with most pronounced atrial cardiomyopathy stages. Importantly, we could not identify a specific subgroup based on atrial cardiomyopathy parameters, which did not show an overall benefit from early rhythm control. Thus, early rhythm control should be considered in all patients with recently diagnosed AF regardless of the severity of atrial cardiomyopathy, including large LA dimension >5 cm.^1^ The modality of ERC depending on atrial cardiomyopathy severity is still a matter of debate. AF ablation is more effective than drug therapy, independent of atrial cardiomyopathy stage. Both strategies might be less effective in patients with more severe atrial cardiomyopathy due to more atrial fibrosis, more extra pulmonary triggers, and a higher degree of electrical remodelling. Therefore, rhythm monitoring after initiation of ERC should be performed more carefully in patients with more severe atrial cardiomyopathy. Of note, we can show that the duration of AF (paroxAF vs. persAF) contributes to more severe stages of atrial cardiomyopathy, which supports the concept that AF burden is an accelerator of atrial cardiomyopathy.^1^ Thus, further studies are needed to assess the pure impact of atrial cardiomyopathy severity in non-AF patients on clinical outcome, in particular, on stroke and cognitive function.

### Limitations

The EAST-AFNET 4 trial was a randomized, multicentre controlled trial. It was not sufficiently powered for this subgroup analysis. However, this analysis enabled a direct comparison of the effect of early rhythm control and atrial cardiomyopathy parameters. Nevertheless, not all parameters were determined in all individual patients, and therefore, subgroups were small to assess the impact of each individual atrial cardiomyopathy component. The availability of the parameter of atrial cardiomyopathy was 86% for LA-diameter, 57% for NT-proBNP, and 43% for PR interval (due to AF in ECG at baseline), which might have biased the results. The PQ interval was used to assess the impact of electrophysiological changes on outcome. Nevertheless, P-wave duration, which might be a better parameter to define changes in conduction velocities, was not available in the EAST-AFNET trial. A few patients were taking antiarrhythmic drugs before initialization (betablocker 65.3%, potassium channel blocker 2.3%, sodium channel blocker 3.4%), which might have influenced absolute PR-intervals. Furthermore, EAST-AFNET 4 did not use ECG monitoring throughout the trial. Left atrial volume index provides more detailed information compared to LA size. LA volume was not available for this analysis. Due to small size of the group with high severity atrial cardiomyopathy, we merged the group with intermediate phenotype for interaction analysis.

This analysis was limited to AF pattern and could not include AF burden. Patients were not entirely treatment-naïve at the time of randomization. While randomization eliminated biases between treatment groups, selection biases between AF patterns or other means cannot fully be excluded. All analyses are hypothesis-generating.

## Conclusions

Different signs indicating atrial cardiomyopathy can commonly be detected in patients with recently diagnosed AF and comorbidities, substantiating the concept that atrial cardiomyopathy often precedes AF in patients (central illustration). Patients with advanced stages of atrial cardiomyopathy had a higher rate of primary outcome events and more recurrent AF. Nevertheless, early rhythm control therapy seems to retain its efficacy across the spectrum of atrial cardiomyopathy severities. Further research is needed focusing on severe stages of atrial cardiomyopathy, but there seems to be no atrial cardiomyopathy-based reason to withhold rhythm control therapy in these patients.

## Supplementary Material

euaf256_Supplementary_Data

## Data Availability

The data that support the findings of this study are available from AFNET Münster (Germany) on reasonable request.

## Abbreviations:

ACS: acute coronary syndrome
ANGPT2: angiopoietin-2
AF: atrial fibrillation
AFNET: atrial fibrillation network
BMP-10: bone morphogenetic protein 10
EAST-AFNET 4 trial: early treatment of atrial fibrillation for stroke prevention trial
HF: heart failure
IRR: incidence rate ratio
LVEF: left ventricular ejection fraction
Parox AF: paroxysmal atrial fibrillation
Pers AF: persistent atrial fibrillation
