# Supplementary material for: Prevalence and severity of atrial cardiomyopathy in patients with recently diagnosed atrial fibrillation and stroke risk factors and its association with early rhythm control: a secondary analysis of EAST-AFNET 4
Source: Europace. 2025 Oct 8;27(10):euaf256. doi: 10.1093/europace/euaf256 (PMC12559886; doi:10.1093/europace/euaf256)
Supplement: euaf256_Supplementary_Data [file euaf256_supplementary_data.docx]

**Supplemental Figure 1:**

**A**
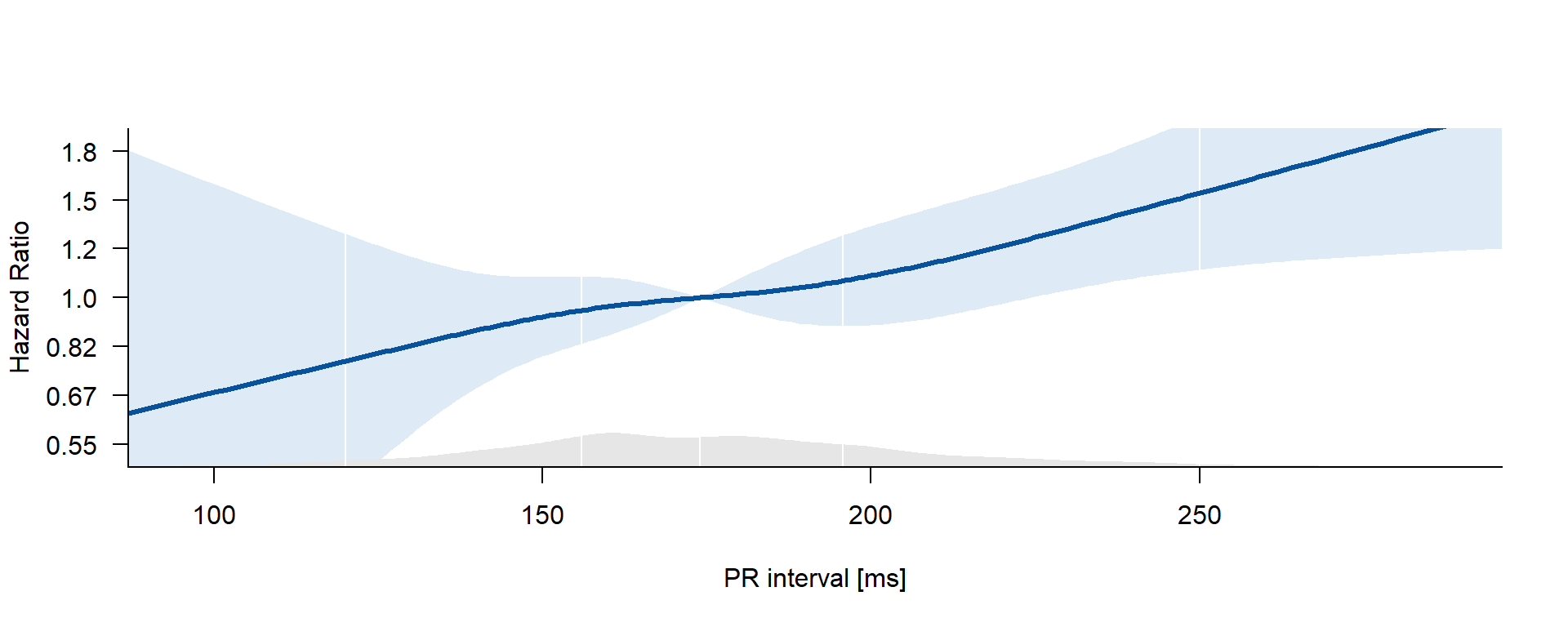


**B**
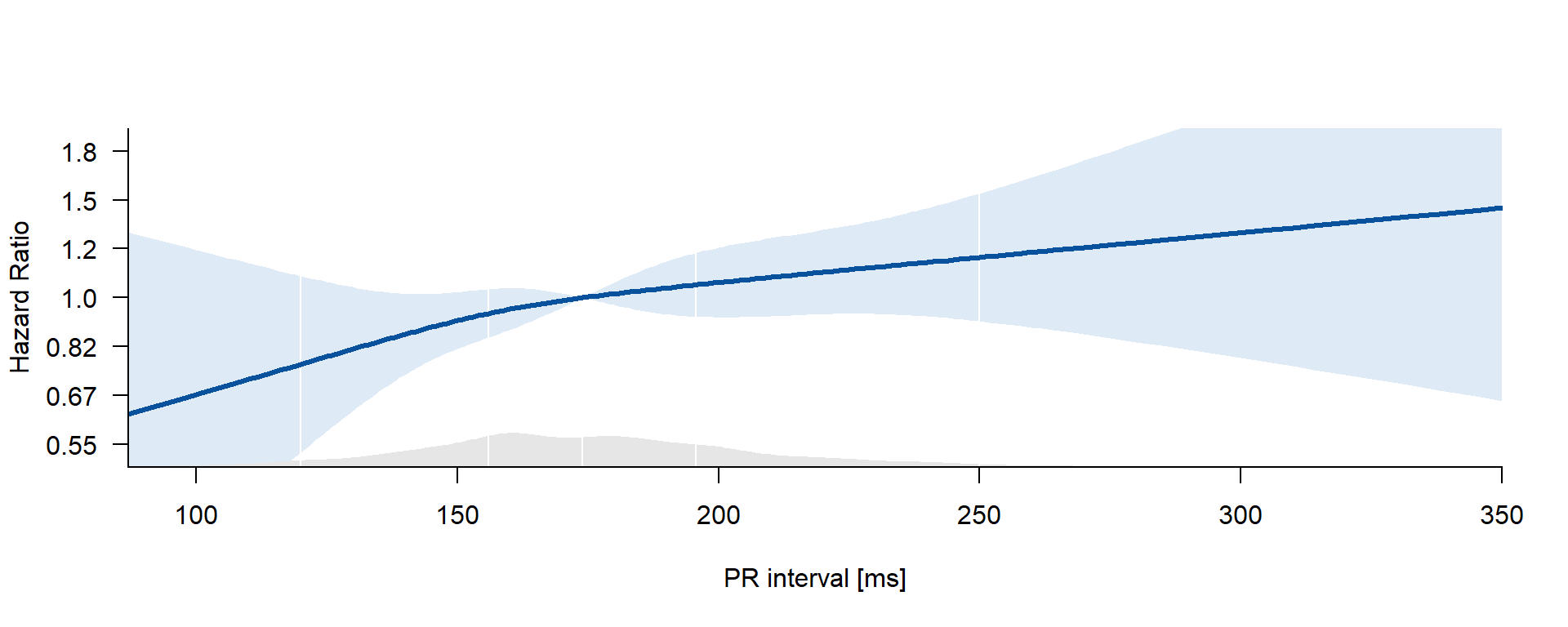


**PR interval and Outcome.** First primary outcome (A), a composite of cardiovascular death, stroke, or unplanned hospitalization for heart failure or acute coronary syndrome, and recurrent AF (B) is associacted with PR interval shown as Hazard Ratio (HR) from Cox regression in this study population. .**Supplemental Figure 2:**

**A**
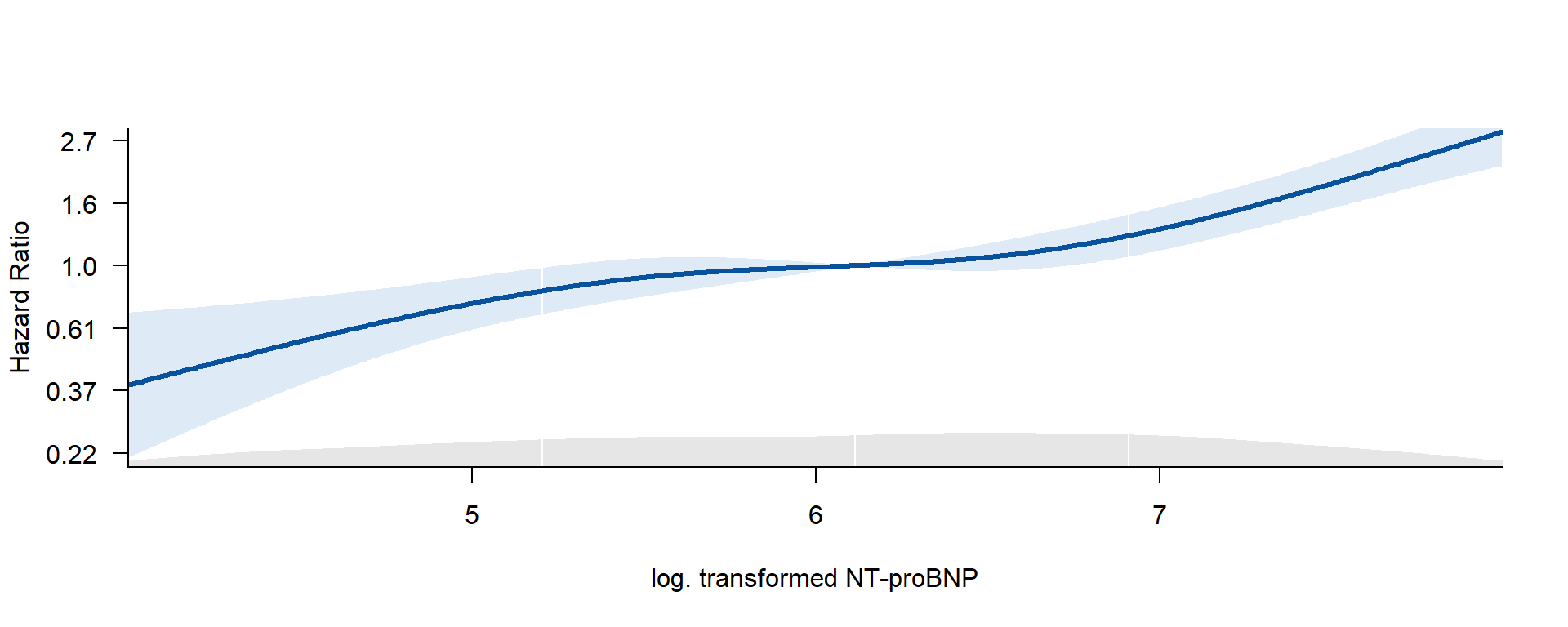
**B**
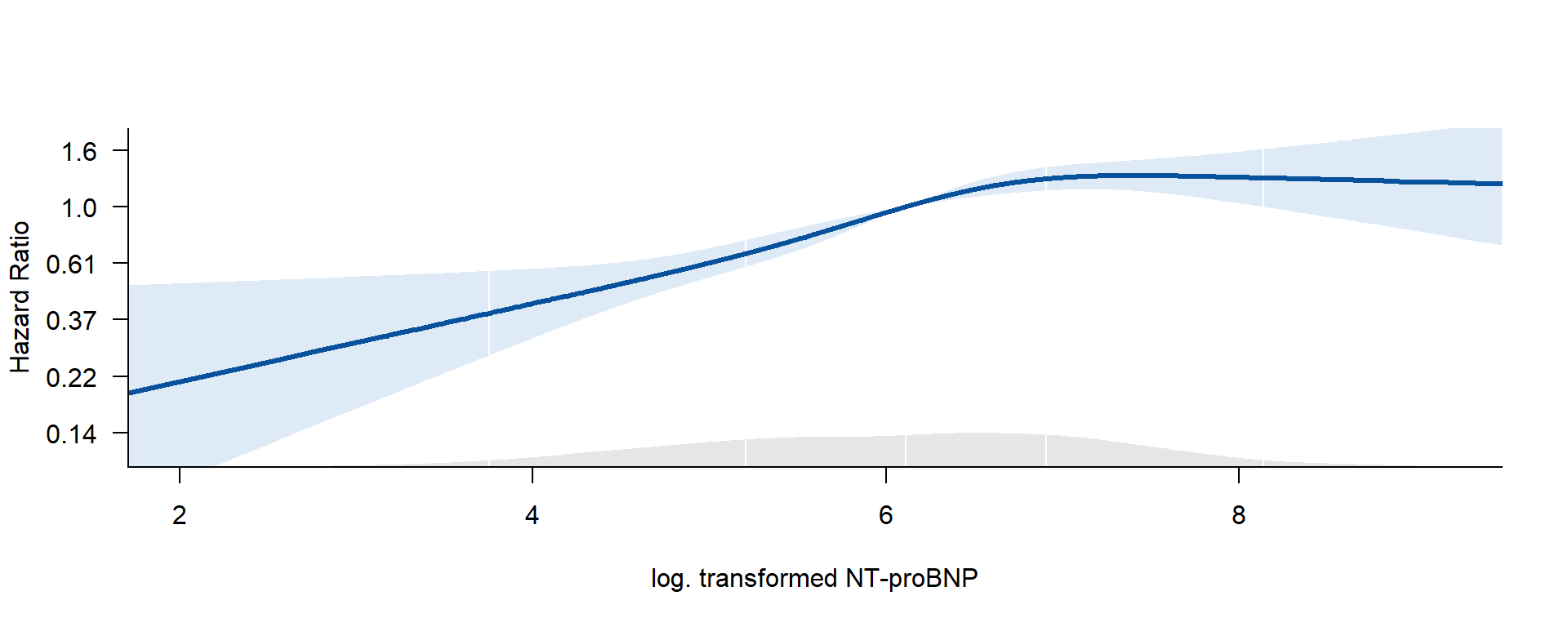


**NT-proBNP and Outcome.** First primary outcome (A), a composite of cardiovascular death, stroke, or unplanned hospitalization for heart failure or acute coronary syndrome, and recurrent AF (B) is associacted with NT-proBNP shown as Hazard Ratio (HR) from Cox regression in this study population. .

**Supplemental Figure 3:**


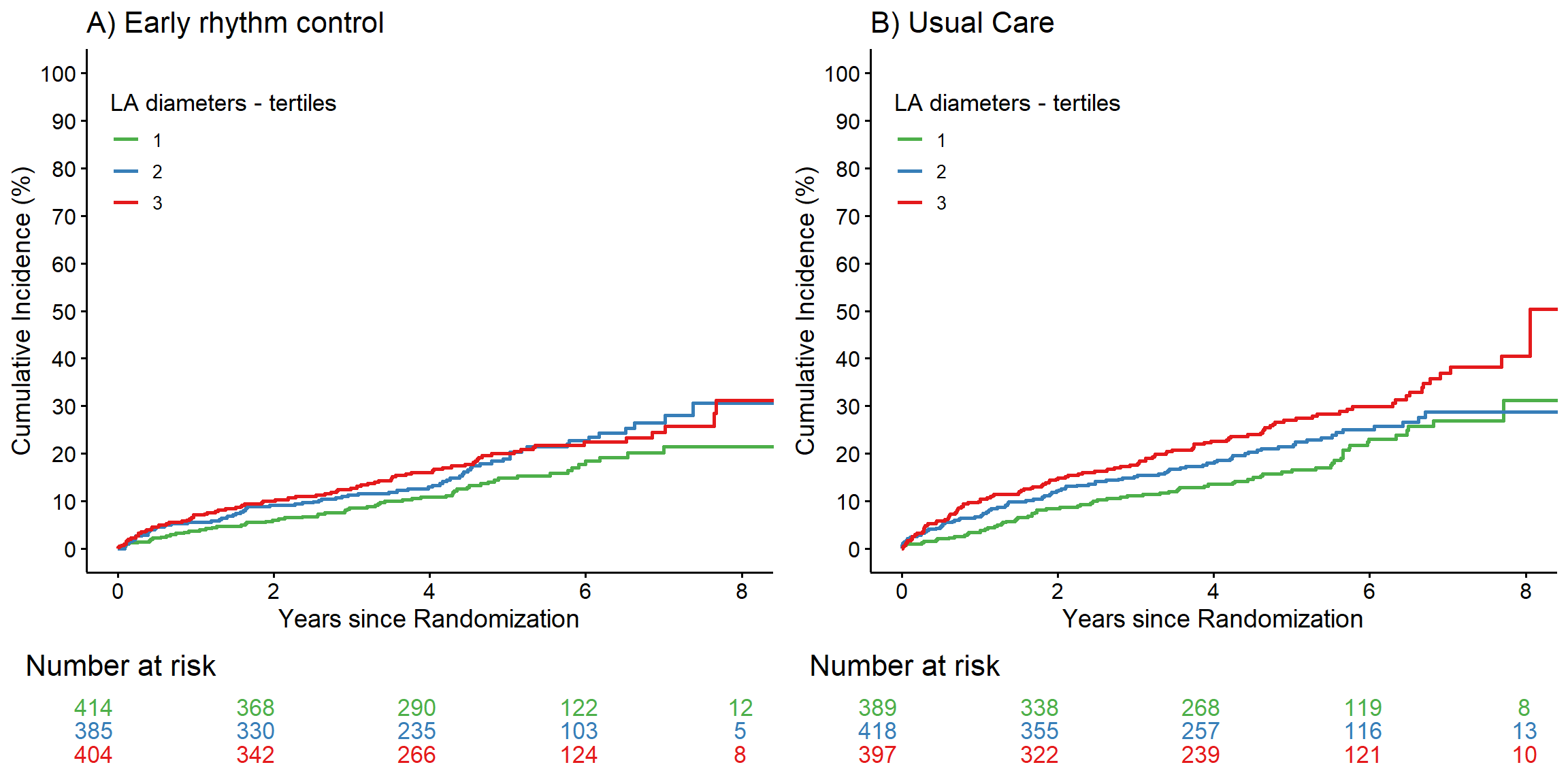


**S-Figure 3**: Aalen-Johnsen Cumulative Curves for first primary outcome, a composite of cardiovascular death, stroke, or unplanned hospitalization for heart failure or acute coronary syndrome, dependent on LA size in tertiles stratified by treatment group (A, Early rhythm control and B, Usual care).

Classification of atrial cardiomyopathy by LA-diameter, NT-proBNP and PR-interval:
 none: all parameter in lower tertile
 mild: all combination in lower and middle tertile
 intermediate: one or two parameter in the upper tertile
 severe: all parameter in upper tertile

**Supplemental Figure 4:**


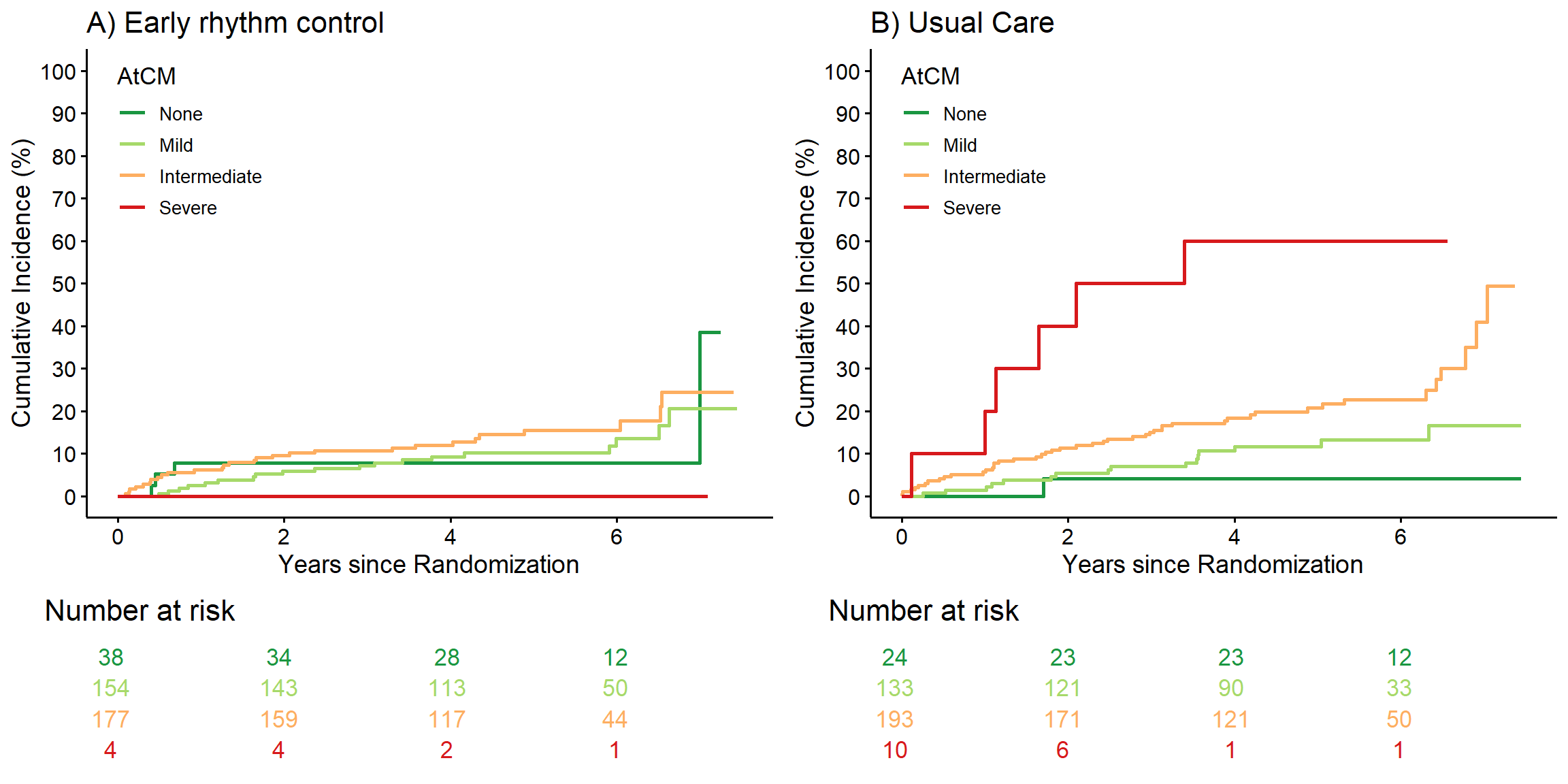


**S-Figure 4**: (A) Aalen-Johnsen Cumulative Curves for first primary outcome, a composite of cardiovascular death, stroke, or unplanned hospitalization for heart failure or acute coronary syndrome, dependent on severity of atrial cardiomyopathyAtrial cardiomyopathy stratified to treatment group (A, Early rhythm control and B, Usual care).

Classification of atrial cardiomyopathy by LA-diameter, NT-proBNP and PR-interval:
 none: all parameter in lower tertile
 mild: all combination in lower and middle tertile
 intermediate: one or two parameter in the upper tertile
 severe: all parameter in upper tertile

**Supplemental Figure 5:**


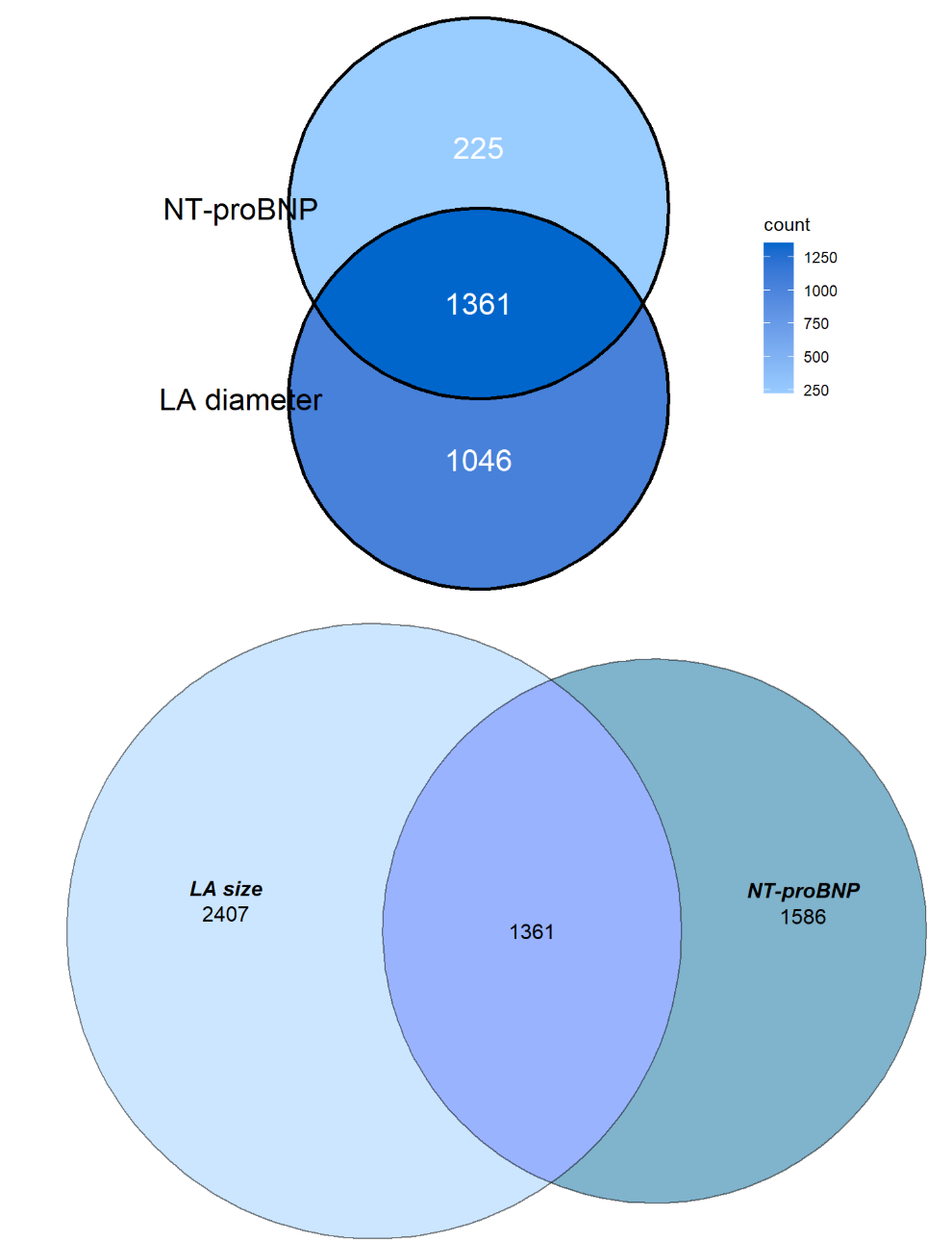


1361 patients have values for the two parameters (LA size by gender, NT-proBNP).

Classification of atrial cardiomyopathy by LA-diameter, NT-proBNP (and without PR-interval):
 none: lower tertile of LA size and NT-proBNP
 mild: all combination of LA size and NT-proBNP in lower and middle tertile
 intermediate: LA size or NT-proBNP in the upper tertile
 severe: upper Tertile of LA size and NT-proBNP

**Supplemental Figure 6:**


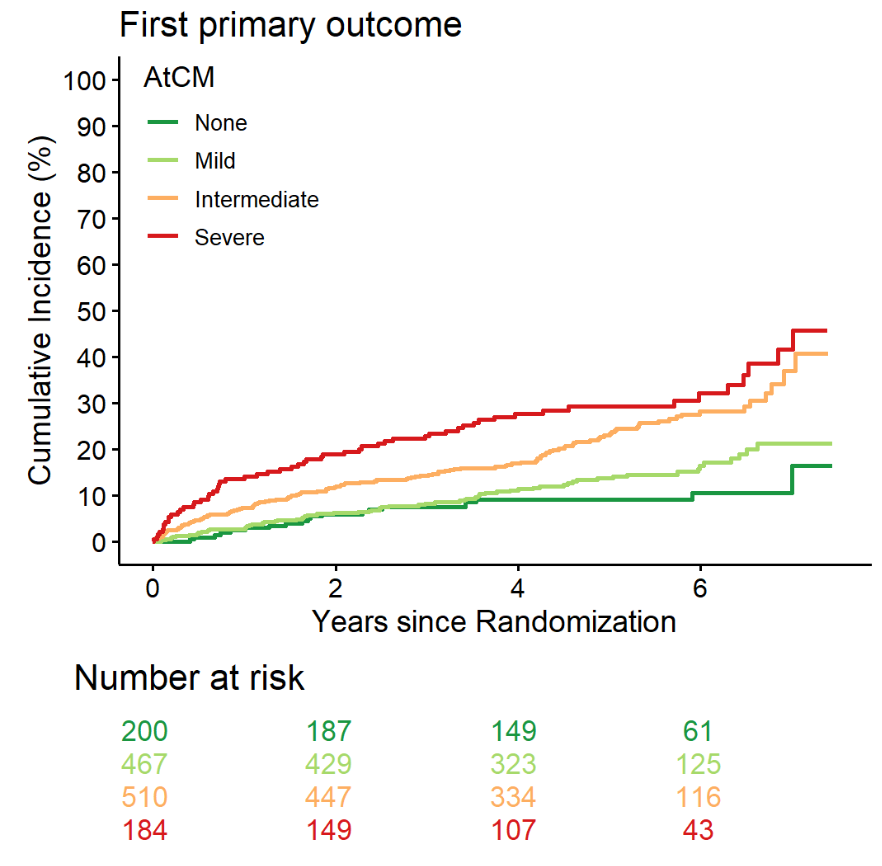


**
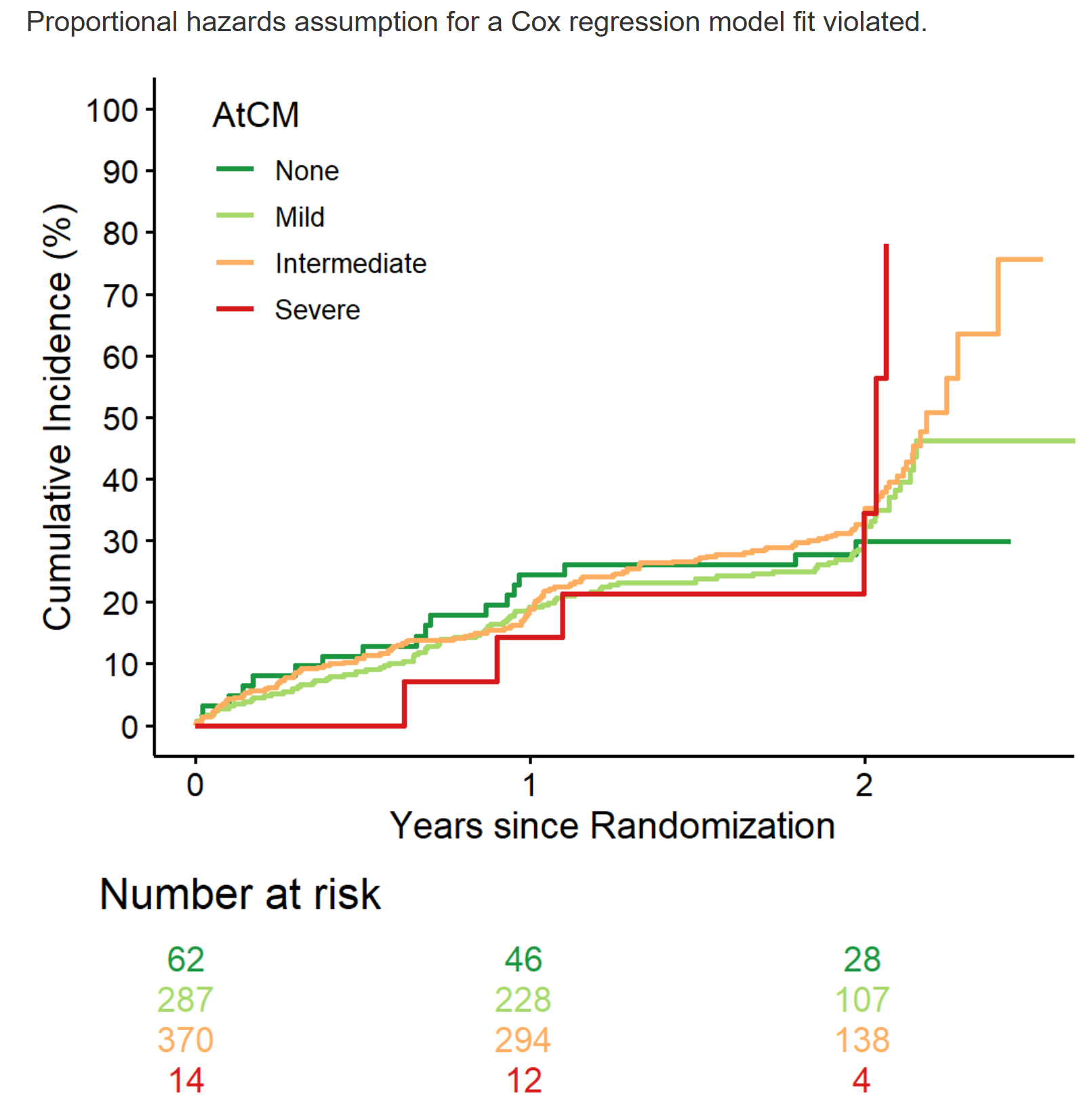
**

**S-Figure 4**: (A) Aalen-Johnsen Cumulative Curves for first primary outcome dependent on severity of atrial cardiomyopathy defined by LA-diameter and NT-proBNP.

Classification of atrial cardiomyopathy by LA-diameter, NT-proBNP (and without PR-interval):
 none: lower tertile of LA size and NT-proBNP
 mild: all combination of LA size and NT-proBNP in lower and middle tertile
 intermediate: LA size or NT-proBNP in the upper tertile
 severe: upper Tertile of LA size and NT-proBNP

**S-Table 1. Baseline characteristics of patients stratified by atrial cardiomyopathy severity³.**

| **Characteristic** | **Overall** N = 733^1^ | **Atrial cardiomyopathy (LA-diameter, NT-proBNP, PR-Interval)^3^** | | | | **p-value**^2^ |
| --- | --- | --- | --- | --- | --- | --- |
|  |  | **None** N = 62 (8.5%)^1^ | **Mild** N = 287 (39%)^1^ | **Intermediate** N = 370 (50%)^1^ | **Severe** N = 14 (1.9%)^1^ |  |
| Age |  |  |  |  |  | 0.006 |
| Mean (SD) | 70 (8) | 68 (8) | 70 (9) | 71 (8) | 75 (6) |  |
| Median (Q1, Q3) | 71 (66, 76) | 69 (66, 75) | 70 (65, 76) | 71 (66, 76) | 78 (69, 79) |  |
| Min, Max | 39, 94 | 49, 83 | 39, 94 | 46, 91 | 63, 83 |  |
| Gender |  |  |  |  |  | 0.13 |
| Female | 377/733 (51%) | 34/62 (55%) | 159/287 (55%) | 175/370 (47%) | 9/14 (64%) |  |
| Male | 356/733 (49%) | 28/62 (45%) | 128/287 (45%) | 195/370 (53%) | 5/14 (36%) |  |
| Body Mass Index [kg/m²] |  |  |  |  |  | 0.012 |
| Mean (SD) | 29.1 (5.2) | 27.3 (4.4) | 28.9 (5.5) | 29.5 (5.1) | 28.9 (4.0) |  |
| Median (Q1, Q3) | 28.7 (25.5, 31.6) | 26.3 (24.5, 30.5) | 28.1 (25.4, 31.3) | 29.2 (26.1, 32.4) | 27.7 (25.8, 32.8) |  |
| Min, Max | 15.9, 53.1 | 19.1, 43.4 | 18.5, 52.6 | 15.9, 53.1 | 22.9, 35.2 |  |
| Diabetes mellitus | 177/733 (24%) | 11/62 (18%) | 64/287 (22%) | 101/370 (27%) | 1/14 (7.1%) | 0.10 |
| CAD | 113/733 (15%) | 7/62 (11%) | 41/287 (14%) | 61/370 (16%) | 4/14 (29%) | 0.37 |
| LVEF < 50% | 47/731 (6.4%) | 1/62 (1.6%) | 15/286 (5.2%) | 30/369 (8.1%) | 1/14 (7.1%) | 0.24 |
| NYHA classification |  |  |  |  |  | <0.001 |
| 0 | 548/733 (75%) | 56/62 (90%) | 221/287 (77%) | 266/370 (72%) | 5/14 (36%) |  |
| I | 73/733 (10.0%) | 3/62 (4.8%) | 31/287 (11%) | 39/370 (11%) | 0/14 (0%) |  |
| II | 91/733 (12%) | 3/62 (4.8%) | 30/287 (10%) | 52/370 (14%) | 6/14 (43%) |  |
| III | 21/733 (2.9%) | 0/62 (0%) | 5/287 (1.7%) | 13/370 (3.5%) | 3/14 (21%) |  |
| Type of atrial fibrillation |  |  |  |  |  | <0.001 |
| First episode | 277/733 (38%) | 28/62 (45%) | 105/287 (37%) | 140/370 (38%) | 4/14 (29%) |  |
| Paroxysmal | 369/733 (50%) | 31/62 (50%) | 161/287 (56%) | 172/370 (46%) | 5/14 (36%) |  |
| Persistent | 87/733 (12%) | 3/62 (4.8%) | 21/287 (7.3%) | 58/370 (16%) | 5/14 (36%) |  |
| Median days since AF diagnosis (IQR) |  |  |  |  |  | 0.97 |
| Mean (SD) | 79 (92) | 78 (97) | 81 (95) | 78 (89) | 70 (97) |  |
| Median (Q1, Q3) | 46 (10, 113) | 43 (12, 91) | 47 (11, 121) | 46 (9, 112) | 22 (6, 143) |  |
| Min, Max | 0, 618 | 0, 403 | 0, 618 | 0, 415 | 1, 299 |  |
| Absence of atrial fibrillation symptoms | 215/685 (31%) | 18/59 (31%) | 93/265 (35%) | 99/348 (28%) | 5/13 (38%) | 0.24 |
| Planned therapy for rhythm control at baseline |  |  |  |  |  | 0.013 |
| AAD | 361/733 (49.25%) | 34/62 (54.84%) | 152/287 (52.96%) | 170/370 (45.95%) | 5/14 (35.71%) |  |
| Ablation | 13/733 (1.77%) | 4/62 (6.45%) | 1/287 (0.35%) | 8/370 (2.16%) | 0/14 (0.00%) |  |
| None | 359/733 (48.98%) | 24/62 (38.71%) | 134/287 (46.69%) | 192/370 (51.89%) | 9/14 (64.29%) |  |
| AAD= antiarrhythmic drug; CAD = coronary artery disease; LVEF=left ventricular ejection fraction.  ^1^ Mean (SD) or Frequency with no./total no. (%) | | | | | | |
| ^2^ p-values resulting from mixed linear regression models for metric variables and mixed (multinomial or ordinal) logistic regression models for categorical variables. For categorical variables with more than two categories (not ordinal) Pearson’s Chi-squared Test or Fisher’s Exact Test was used.  ³ Classification of atrial cardiomyopathy by LA-diameter, NT-proBNP and PR-interval:  none: all parameter in lower tertile  mild: all combination in lower and middle tertile  intermediate: one or two parameter in the upper tertile  severe: all parameter in upper tertile | | | | | | |

**Supplemental Table 2.** Safety outcomes stratified tertiles of left atrial size.

|  | **Early rhythm control** | | | **Usual care** | | |  |
| --- | --- | --- | --- | --- | --- | --- | --- |
|  | **T1** | **T2** | **T3** | **T1** | **T2** | **T3** | **p-value interaction** |
| **n** | 414 | 385 | 404 | 389 | 418 | 397 |  |
| **Primary composite safety outcome** | 63 (15.2) | 59 (15.3) | 80 (19.8) | 58 (14.9) | 65 (15.6) | 79 (19.9) | 0.911 |
| **Stroke** | 13 ( 3.1) | 9 ( 2.3) | 13 ( 3.2) | 19 ( 4.9) | 21 ( 5.0) | 20 ( 5.0) | 0.978 |
| **Death** | 37 ( 8.9) | 37 ( 9.6) | 47 (11.6) | 41 (10.5) | 47 (11.2) | 59 (14.9) | 0.78 |
| **Serious adverse event of special interest related to rhythm control therapy** | 16 ( 3.9) | 17 ( 4.4) | 27 ( 6.7) | 6 ( 1.5) | 4 ( 1.0) | 6 ( 1.5) | 0.378 |

**Supplemental Table 3.** **Distribution of atrial cardiomyopathy (LA size & NTproBNP)**

| **atrCM** | **Early rhythm control** | **Usual care** | **Total** |
| --- | --- | --- | --- |
| **None** | 38 (10%) | 24 (6.7%) | 62 (8.5%) |
| **Mild** | 154 (41%) | 133 (37%) | 287 (39%) |
| **Intermediate** | 177 (47%) | 193 (54%) | 370 (50%) |
| **Severe** | 4 (1.1%) | 10 (2.8%) | 14 (1.9%) |
| **Total** | 373 (100%) | 360 (100%) | 733 (100%) |

Classification of atrial cardiomyopathy by LA-diameter, NT-proBNP (and without PR-interval):
 none: lower tertile of LA size and NT-proBNP
 mild: all combination of LA size and NT-proBNP in lower and middle tertile
 intermediate: LA size or NT-proBNP in the upper tertile
 severe: upper Tertile of LA size and NT-proBNP

**S-Table 4 Baseline characteristics per atrial cardiomyopathy(LA size & NTproBNP)**

| **Characteristic** | **Overall (N=1361)** | **None (N=200, 15%)** | **Mild (N=467, 34%)** | **Intermediate (N=510, 37%)** | **Severe (N=184, 14%)** | **p-value** |
| --- | --- | --- | --- | --- | --- | --- |
| **Age - Mean (SD)** | 70 (8) | 69 (8) | 70 (8) | 71 (8) | 71 (8) | <0.001 |
| **Age - Median (Q1, Q3)** | 71 (66,76) | 69 (65,75) | 71 (66,76) | 72 (67,76) | 72 (67,77) |  |
| **Age - Min, Max** | 39, 94 | 48, 91 | 39, 94 | 42, 91 | 48, 88 |  |
| **Gender - Female** | 616/1361 (45%) | 91/200 (46%) | 222/467 (48%) | 219/510 (43%) | 84/184 (46%) | 0.63 |
| **Gender - Male** | 745/1361 (55%) | 109/200 (55%) | 245/467 (52%) | 291/510 (57%) | 100/184 (54%) |  |
| **BMI - Mean (SD)** | 29.4 (5.4) | 28.2 (4.8) | 29.2 (5.2) | 29.6 (5.4) | 30.4 (6.3) | <0.001 |
| **BMI - Median (Q1, Q3)** | 28.7 (25.5,32.4) | 27.5 (25.0,30.9) | 28.7 (25.6,31.7) | 29.2 (25.6,33.0) | 29.4 (25.9,33.6) |  |
| **BMI - Min, Max** | 15.9, 58.2 | 15.9, 51.3 | 16.8, 52.6 | 16.6, 53.1 | 19.0, 58.2 |  |
| **Diabetes mellitus** | 339/1361 (25%) | 39/200 (20%) | 121/467 (26%) | 133/510 (26%) | 46/184 (25%) | 0.23 |
| **CAD** | 222/1361 (16%) | 23/200 (12%) | 73/467 (16%) | 89/510 (17%) | 37/184 (20%) | 0.12 |
| **LVEF < 50%** | 171/1352 (13%) | 3/198 (1.5%) | 35/466 (7.5%) | 80/505 (16%) | 53/183 (29%) | <0.001 |
| **NYHA 0** | 922/1361 (68%) | 176/200 (88%) | 336/467 (72%) | 326/510 (64%) | 84/184 (46%) | <0.001 |
| **NYHA I** | 156/1361 (11%) | 14/200 (7%) | 47/467 (10%) | 67/510 (13%) | 28/184 (15%) |  |
| **NYHA II** | 238/1361 (17%) | 8/200 (4%) | 71/467 (15%) | 99/510 (19%) | 60/184 (33%) |  |
| **NYHA III** | 45/1361 (3.3%) | 2/200 (1%) | 13/467 (2.8%) | 18/510 (3.5%) | 12/184 (6.5%) |  |
| **AF Type - First episode** | 475/1361 (35%) | 81/200 (41%) | 171/467 (37%) | 176/510 (35%) | 47/184 (26%) | <0.001 |
| **AF Type - Paroxysmal** | 490/1361 (36%) | 109/200 (55%) | 208/467 (45%) | 143/510 (28%) | 30/184 (16%) |  |
| **AF Type - Persistent** | 396/1361 (29%) | 10/200 (5%) | 88/467 (19%) | 191/510 (37%) | 107/184 (58%) |  |
| **Median days since AF diagnosis - Mean (SD)** | 86 (185) | 79 (102) | 81 (164) | 93 (239) | 89 (121) | 0.50 |
| **Median days since AF diagnosis - Median (Q1,Q3)** | 44 (9,113) | 38 (9,104) | 39 (8,106) | 50 (10,118) | 46 (13,121) |  |
| **Median days since AF diagnosis - Min,Max** | 0,4586 | 0,426 | 0,2739 | 0,4586 | 0,1148 |  |
| **Absence of AF symptoms** | 429/1277 (34%) | 58/189 (31%) | 156/434 (36%) | 161/478 (34%) | 54/176 (31%) | 0.49 |
| **Planned therapy - AAD** | 647/1361 (47.5%) | 112/200 (56%) | 233/467 (49.9%) | 215/510 (42.2%) | 87/184 (47.3%) | <0.001 |
| **Planned therapy - Ablation** | 45/1361 (3.3%) | 6/200 (3%) | 5/467 (1.1%) | 21/510 (4.1%) | 13/184 (7.1%) |  |
| **Planned therapy - None** | 669/1361 (49.2%) | 82/200 (41%) | 229/467 (49%) | 274/510 (53.7%) | 84/184 (45.7%) |  |

1 Mean (SD) or Frequency with no./total no. (%)

2 p-values resulting from mixed linear regression models for metric variables and mixed (multinomial or ordinal) logistic regression models for categorical variables. For categorical variables with more than two categories (not ordinal) Pearson’s Chi-squared Test or Fisher’s Exact Test was used.

Classification of atrial cardiomyopathy by LA-diameter, NT-proBNP (and without PR-interval):
 none: lower tertile of LA size and NT-proBNP
 mild: all combination of LA size and NT-proBNP in lower and middle tertile
 intermediate: LA size or NT-proBNP in the upper tertile
 severe: upper Tertile of LA size and NT-proBNP

**S-Table 5. Primary outcome for severy of atrial cardiomyopathy based on LA-size and NT-proBNP**

**Hazard Ratios per atrial Cardiomyopathy**

| **Atrial cardiomyopathy** | **HR per SD** | **95%-CI** | **p value** |
| --- | --- | --- | --- |
| **None** | Ref |  |  |
| **Mild** | 1.59 | [0.62 , 4.08] | 0.339 |
| **Intermediate** | 2.8 | [1.12 , 6.99] | 0.027 |
| **Severe** | 7.97 | [2.32 , 27.37] | 0.001 |

**Note:**
Anova test p-value: 0.0005

Classification of atrial cardiomyopathy by LA-diameter, NT-proBNP (and without PR-interval):
 none: lower tertile of LA size and NT-proBNP
 mild: all combination of LA size and NT-proBNP in lower and middle tertile
 intermediate: LA size or NT-proBNP in the upper tertile
 severe: upper Tertile of LA size and NT-proBNP

**S-Table 6. AF recurrence for severity of atrial cardiomyopathy based on LA-size and NT-proBNP**

| **Atrial cardiomyopathy** | **HR per SD** | **95%-CI** | **p value** |
| --- | --- | --- | --- |
| **None** | Ref |  |  |
| **Mild** | 1.49 | [1.12 , 1.99] | 0.006 |
| **Intermediate** | 1.96 | [1.48 , 2.59] | <0.001 |
| **Severe** | 2.75 | [2.01 , 3.78] | <0.001 |

**Note:** Anova test p-value: 0.0005

**Cox Regression for Recurrent AF ~ AtrCM + frailty term**

| **Atrial cardiomyopathy** | **HR per SD** | **95%-CI** | **p value** |
| --- | --- | --- | --- |
| **None/Mild** | Ref |  |  |
| **Intermediate/Severe** | 2.14 | [1.7 , 2.7] | <0.001 |

**Note:** Anova test p-value: 0

Classification of atrial cardiomyopathy by LA-diameter, NT-proBNP (and without PR-interval):
 none: lower tertile of LA size and NT-proBNP
 mild: all combination of LA size and NT-proBNP in lower and middle tertile
 intermediate: LA size or NT-proBNP in the upper tertile
 severe: upper Tertile of LA size and NT-proBNP
